# Practical Notes on Diagnosis and Treatment

**Published:** 1907-04-20

**Authors:** 


### Practical Notes on Diagnosis and Treatment.

**Leucorrhœa.**

A solution of alum, 10 to 20 grains to the ounce, is a useful injection in cases of leucorrhœa.

**Deafness in Congenital Syphilis.**

We know of no form of ear disease other than that due to syphilis in which, without any obvious destruction of parts, complete and permanent deafness in both ears can be brought about in the course of a few weeks. But this is not a rare occurrence in the subjects of inherited syphilis.—*Mr. Jonathan Hutchinson.*

**Bruit in Aneurism.**

With regard to abdominal aneurism and all other aneurisms, I want to say that a bruit is one of the least important and most misleading of diagnostic points. There may be aneurism without bruit, and again, a bruit over a tumour does not necessarily mean that the tumour is aneurismal. Further, I have met with cases both of thrill and bruit in aortic pulsation which were not cases of aneurism.—*Sir Douglas Powell.*

**Gonorrhœa.**

In the early stages of this disease injections should never be employed, and in the free secretion stage most cases can be cured by simply administering the oleo-balsams, keeping the patient's bowels open, and forbidding alcohol. In neglected and chronic cases injections are most valuable, and among those to be recommended are a 3 per cent. watery solution of ichthyol, or permanganate of zinc., ½ grain or more to the ounce. One of the best is: Zinc sulphate grs. 3, tinct. catechu ♏10., tinct. opium ♏5, glycerine ♏15, water to 1 oz.—*Mr. Albert Carless.*

**Optic Neuritis.**

The first and chief significance of optic neuritis is the presence of organic disease. If you can exclude a blood state or a constitutional condition, you may feel sure that there is organic disease within the skull or orbit. But neuritis is important, not only for diagnosis, but also for prognosis. Commencing subsidence of the neuritis may be the first indication of commencing subsidence of a morbid process in the brain, and persistence of the neuritis may be the indication of persistence of disease in the brain even when, for the time being, all other symptoms have passed away.—*Sir Wm. Gowers.*

**Ringworm of the Scalp.**

After cutting the hair short, the head should be washed every morning with soft soap and water, and then the following ointment is applied on linen, the whole being covered with gutta-percha tissue and secured by a skull-cap:—Salicylic acid grs. 10, chrysophanic acid grs. xxv., ichthyol 30 per cent., vaseline to ℥j. This is repeated for four consecutive days, when the ointment as above is replaced by a 20 per cent. ichthyol ointment. Then, after a further four days, the first ointment is substituted, and this alternation is continued. As a rule a cure can be effected in from three to six weeks.—*Professor Unna.*

**Pityriasis Versicolor.**

Turpentine will promptly remove the evidences of this disease from the skin. To prevent reappearance of the spots the clothing should be disinfected.

**Hæmorrhoids.**

The following is a useful application:—Calomel ʒss, hydrochloride of morphine grs. iiss, bismuth subnitrate ʒvj, glycerine ʒij, vaseline ʒvj. Make an ointment and apply freely.

**Chronic Gonorrhœa.**

The following may be applied to the urethra by means of a flexible French bougie:—Yellow oxide of mercury 4 grains, lanoline and white vaseline, of each, half an ounce.

**An Anodyne Lotion.**

Chloroform liniment, to which is added menthol in the proportion of one dram to an ounce of the liniment, may be successfully applied as a lotion for the relief of neuralgic pains.

**Epididymitis.**

The following has given good results in acute epididymitis:—Solution of acetate of morphine ʒiss, chloral hydrate ʒiss, ammonium bromide ʒij, syrup of tolu ʒiv, water to ℥iv. A tablespoonful every three hours.

**Retention of Urine in Hysteria.**

When you hear, in the case of a young girl who is obviously healthy and properly formed, a story of the patient not passing urine, you should at once think of hysteria and should believe that in all probability there is some fraud in the case.—*Sir Dyce Duckworth.*

**Chronic Metritis.**

Chronic metritis is to a large extent, if not at first at any rate afterwards, a constitutional condition, and everything which improves the constitutional state of the patient improves her state as regards the metritis. The two best remedies to give internally are perchloride of mercury and iodide of potassium.—*Dr. J. B. Potter.*

**Alkaline Treatment in Rheumatism.**

In many cases of subacute rheumatism alkalies will succeed when the value of salicylates has become exhausted. But it is essential that full doses should be given, and this can only be determined by noting an alkaline reaction of the urine. Quinine may be usefully combined with the bicarbonate and citrate of potassium.

**Opium in Delirium Tremens.**

Before you decide to give opium you should make sure that the kidneys are sound, as any suspicion in that direction absolutely forbids the use of the drug. If you give opium at all you should give it early and in a large dose—40 to 60 drops of laudanum for example. To begin with 20 drops, then 40, then 60, as the days go on, is a most dangerous practice. Further, as long as you do not improve the patient's nutrition opiates will be in vain. Free feeding is therefore essential.—*Dr. A. B. Duffin.*

